# Characterization and expression profiles of miRNAs in the triploid hybrids of *Brassica napus* and *Brassica rapa*

**DOI:** 10.1186/s12864-019-6001-x

**Published:** 2019-08-14

**Authors:** Libin Zhang, Jun Zou, Shisheng Li, Baoshan Wang, Nadia Raboanatahiry, Maoteng Li

**Affiliations:** 1 College of Life Science and Technology, Huazhong University of Science and Technology, Wuhan, China; 2 College of Plant Science and Technology, Huazhong Agricultural University, Wuhan, China; 3 Hubei Collaborative Innovation Center for the Characteristic Resources Exploitation of Dabie Mountains, Huanggang Normal University, Huanggang, 438000 China; 4 College of Life Science, Shandong Normal University, Jinan, 250000 China

**Keywords:** *B. rapa*, *B. napus*, Hybrid, MicroRNAs, Small RNA sequencing

## Abstract

**Background:**

Polyploidy provides a means of interspecific genome transfer to incorporate preferable traits from progenitor to progeny. However, few studies on miRNA expression profiles of interspecific hybrids of *B. napus* (AnAnCnCn) and *B. rapa* (ArAr) have been reported.

**Results:**

Here, we apply small RNA sequencing to explore miRNA expression patterns between *B. napus*, *B. rapa* and their F1 hybrid. Bioinformatics analysis identified 376, 378, 383 conserved miRNAs and 82, 76, 82 novel miRNAs in *B. napus*, *B. rapa* and the F1 hybrid, respectively. Moreover, 213 miRNAs were found to be differentially expressed between *B. napus*, *B. rapa* and the F1 hybrid. The present study also shows 211 miRNAs, including 77 upregulated and 134 downregulated miRNAs, to be nonadditively expressed in the F1 hybrid. Furthermore, miRNA synteny analysis revealed high genomic conservation between the genomes of *B. napus*, *B. rapa* and their F1 hybrid, with some miRNA loss and gain events in the F1 hybrid.

**Conclusions:**

This study not only provides useful resources for exploring global miRNA expression patterns and genome structure but also facilitates genetic research on the roles of miRNAs in genomic interactions of *Brassica* allopolyploids.

**Electronic supplementary material:**

The online version of this article (10.1186/s12864-019-6001-x) contains supplementary material, which is available to authorized users.

## Background

Polyploidy has been recognized as an important force in plant evolution as well as in novel species formation [1]. Genome duplication increases available genetic materials and results in stronger drought, disease and insect pest resistance, which enables polyploid species to have greater advantages in natural selection. [2, 3]. By comparing the cytology, molecular biology and molecular genetics of polyploids with their parents, very complex genetic variations, including methylation, gene silencing, gene activation and small RNA changes, have usually been found in the former [4, 5].

MicroRNAs (miRNAs) are a class of non-coding single-stranded RNA molecules encoded by endogenous genes with a length of approximately 22 nucleotides [6, 7]. It has been reported that miRNAs are involved in various stages of plant growth and development by silencing target gene(s), and they can be applied for molecular breeding of crop species through direct molecular modulation of specific traits [8]. For example, miRNA controls multiple signal transduction pathways and participates in plant cell differentiation, flower development and seed formation [9–13]. It has been reported that small RNAs act as a genetic buffer in interspecific hybrids and allopolyploids of *Arabidopsis* [12]. Additionally, multiple small RNAs, including miRNAs, are associated with the molecular mechanism of heterosis in maize [14–17]. Moreover, previous studies showed that various miRNAs were nonadditively expressed in *Brassica* hexaploids compared with their parents [18–21], suggesting a correlation of miRNA expression patterns with many phenotypes in plant allopolyploids. *Brassica napus* is an important polyploid oil crop and the largest oil-bearing crop in China, accounting for 45% of the total output of edible oil [22–26]. *B. rapa belongs to the Brassicaceae* family and is widely planted in China [27–31]. Additionally, *Brassica* species are widely used as model systems to study genomic changes in allopolyploidization. For example, a study of molecular markers and biomass heterosis in the interspecific hybrid between *B. napus* and *B. rapa* has been reported [32, 33]. Overall, natural triploid plants have great potential in polyploid breeding, and introgression of genetic resources of *B. rapa* (AA genome) into *B. napus* will broaden the genetic basis of the latter and have important application significance [34–36]. For instance, interspecific hybridization between *B. napus* and *B. rapa* was found to be an efficient and important method of introgressing *B. rapa* germplasm into *B. napus* [37]. Triploids from crosses of *B. napus* and *B. rapa* are ideal models to assess homologous pairing and recombination occurring between their genomes. Moreover, these triploid hybrids have been used for the production of monosomic addition lines and for gene flow assessment after backcrossing to either parent [38]. The availability of polyploids of *B. napus* and *B. rapa* allows us to explore the effects of polyploidization on global gene expression through more accurate comparisons of triploids and their progenitors. Furthermore, the genomes of *B. napus* and *B. rapa* have been recently sequenced [39, 40], providing available genome information to globally identify miRNAs and analyze miRNA expression patterns among *B. napus*, *B. rapa* and their hybrid.

miRNAs play important roles in the biological and metabolic processes of seeds, including embryogenesis, dormancy and germination [8]. The embryo is a crucial tissue affecting seed development and germination. Although interspecific hybridization between *B. napus* and *B. rapa* has been widely investigated [32], the expression pattern and diversity of embryo miRNAs in triploids remain unclear. The purpose of this study was to investigate the expression profile of miRNAs and their potential targets in triploid hybrids and their progenitors, which may provide vital clues for further and detailed functional studies of embryo miRNAs. In this study, the embryo tissues of triploid hybrid (A^n^A^r^C^n^, 21 days after pollination) and control embryo tissues of *B. napus* and *B. rapa* were isolated for small RNA sequencing. Conserved and novel miRNAs of *B. rapa*, *B. napus* and their hybrid were identified and characterized, and the miRNA expression profiles in the developing embryos of *B. napus*, *B. rapa* and their hybrid were investigated. The results showed 376, 378 and 383 conserved miRNAs and 82, 76 and 82 novel miRNAs in the *B. napus*, *B. rapa* and F1 hybrid, respectively. Moreover, 213 miRNAs were found to be differentially expressed between *B. napus*, *B. rapa* and the F1 hybrid, and 211 miRNAs, including 77 upregulated and 134 downregulated miRNAs, were nonadditively expressed in the F1 hybrid. miRNA synteny analysis revealed some miRNA loss and gain events in the genome of the F1 hybrid. Collectively, this study identified conserved and novel miRNAs and investigated genome-wide miRNA expression profiles between *B. napus*, *B. rapa* and their F1 triploid hybrid, and the findings will be very helpful in promoting research on miRNA roles in the genomic interactions of triploids.

## Materials and methods

### Plant materials

*B. napus* var. Huashuang 3 (Female parent, A^n^A^n^C^n^C^n^, 2n = 38) was obtained from Professor Jiangsheng Wu in Huazhong Agricultural University (Professor Jiangsheng Wu bred Huashuang 3 variety). *B. rapa* var. Tianmen Youcaibai (A^r^A^r^, 2n = 18) is a local rapeseed variety in Hubei province. The triploid hybrid (AnArCn, *n* = 29) was obtained by the hybridization between *B. napus* var. Huashuang 3 (Female parent, A^n^A^n^C^n^C^n^, 2n = 38) and *B. rapa* var. Tianmen Youcaibai (A^r^A^r^, 2n = 18). The developing embryos of these plants (21 days after pollination) were harvested and immediately frozen in liquid nitrogen. Total RNAs were isolated using Trizol reagent (Invitrogen, USA). The isolated RNAs were quantified and put in − 80 °C freezer.

### Small RNAs sequencing

The total RNA of developing embryos of *B. napus*, *B. rapa* and their triploid hybrid (21 days after pollination) was isolated for small RNA sequencing as previously described [41]. The small RNA libraries were sequenced on Illumina HiSeq 2000 platform. The adaptor sequences and low-quality reads were first removed before further bioinformatics analysis. The obtained clean reads were mapped to the non-coding RNA sequences in Rfam database. The mapped reads of snoRNAs, snRNAs, tRNAs, rRNAs and materials containing the poly(A) tail were removed. The filtered sRNAs-seq clean reads were submitted to the NCBI/SRA database with accession number.

### Prediction of miRNAs and targets

The conserved and novel miRNAs of *B. napus*, *B. rapa* and the triploid hybrid were predicted according to published criteria [42]. The sRNAs-seq clean reads of *B. napus*, *B. rapa* and their triploid hybrid were mapped to the genomes of *B. napus* and *B. rapa*, respectively. Mireap (http://sourceforge.net/projects/mireap/) was then used to predict miRNA hairpin structures with published parameters. By alignment with miRbase (http://www.mirbase.org/index.shtml), the miRNAs containing ≤2 nucleotide mismatches to known miRNAs were identified to be conserved miRNAs, while the miRNAs containing > 2 nucleotide mismatches to known miRNAs were considered to be novel miRNAs. The miRNA targets of *B. napus*, *B. rapa* and their triploid hybrid were predicted respectively using psRobot (http://omicslab.genetics.ac.cn/psRobot/.) according to the published rules [43, 44].

### MiRNA differential expression analysis

In order to identify differentially expressed miRNAs between *B. napus*, *B. rapa* and their triploid hybrid, the expression level of miRNAs in *B. napus* or *B. rapa* was set as a control and the up- or down-regulated miRNAs in triploid hybrid were analyzed by edgeR [45]. MiRNA expression quantification was normalized according to the expression of transcript per million (TPM). First, The TPM of the conserved and novel miRNA in *B. napus*, *B. rapa* and their hybrid were first calculated. The fold change values of selected miRNAs were then normalized based on their TPMs. The *P*-value significance threshold was set by the false discovery rate (FDR). For the comparison of miRNA expression level between *B. napus*, *B. rapa* and their hybrid, a fold change of miRNA expression level greater than 2 (with FDR < 0.001 and *P* value < 0.01) was considered as an indication of significant change and the miRNA was considered to be differentially expressed between *B. napus*, *B. rapa* and triploid hybrid. Hierarchical clustering analysis of differentially expressed miRNAs was performed using the Euclidean distance measurement with the R package pheatmap (https://cran.r-project.org/web/packages/pheatmap/index.html).

### Quantitative RT-PCR analysis of differentially expressed miRNAs

To validate the differentially expressed miRNAs between *B. napus*, *B. rapa* and their triploid hybrid, qRT-PCR was used for expression level analysis. For cDNA synthesis, 1 μg of total RNA from *B. napus*, *B. rapa* and their triploid hybrid were reverse-transcribed a RevertAid First Strand cDNA Synthesis Kit (Thermo Scientific). Quantitative RT-PCR reactions were performed using miRNA qRT-PCR kit (#AMPR-0200, GeneCopoeia) with U6 snRNA as the internal control. The comparative CT method was used for the calculation of the relative expression fold changes of miRNAs. All reactions were performed in triplicate with three independent experiments. The primer sequences were listed in Additional file 1: Table S1.

### MiRNA synteny analysis

The synteny among *B. napus*, *B. rapa* and their triploid hybrid was analyzed as previously described [42]. *Brassica napus* genome resource (http://www.genoscope.cns.fr/brassicanapus/) and Brassica Database (http:// brassicadb.org/) were used for miRNA synteny analysis. The 10 flanking protein-coding loci of every miRNA were extracted from *B. napus* and *B. rapa* genomes. BLASTn was used to perform the homology test of miRNAs and their flanking genes and top-5 hits of every miRNA were selected. The miRNAs containing 1 identical upstream or downstream flanking coding genes were identified as syntenic miRNAs among *B. napus*, *B. rapa* and their triploid hybrid. The identified syntenic miRNAs were classified into 4 sets [46], in which the first 3 sets were used for the construction of Circos map [47].

## Results

### Small RNA sequencing of *B. napus*, *B. rapa* and their triploid hybrid

**Fig. 1:**
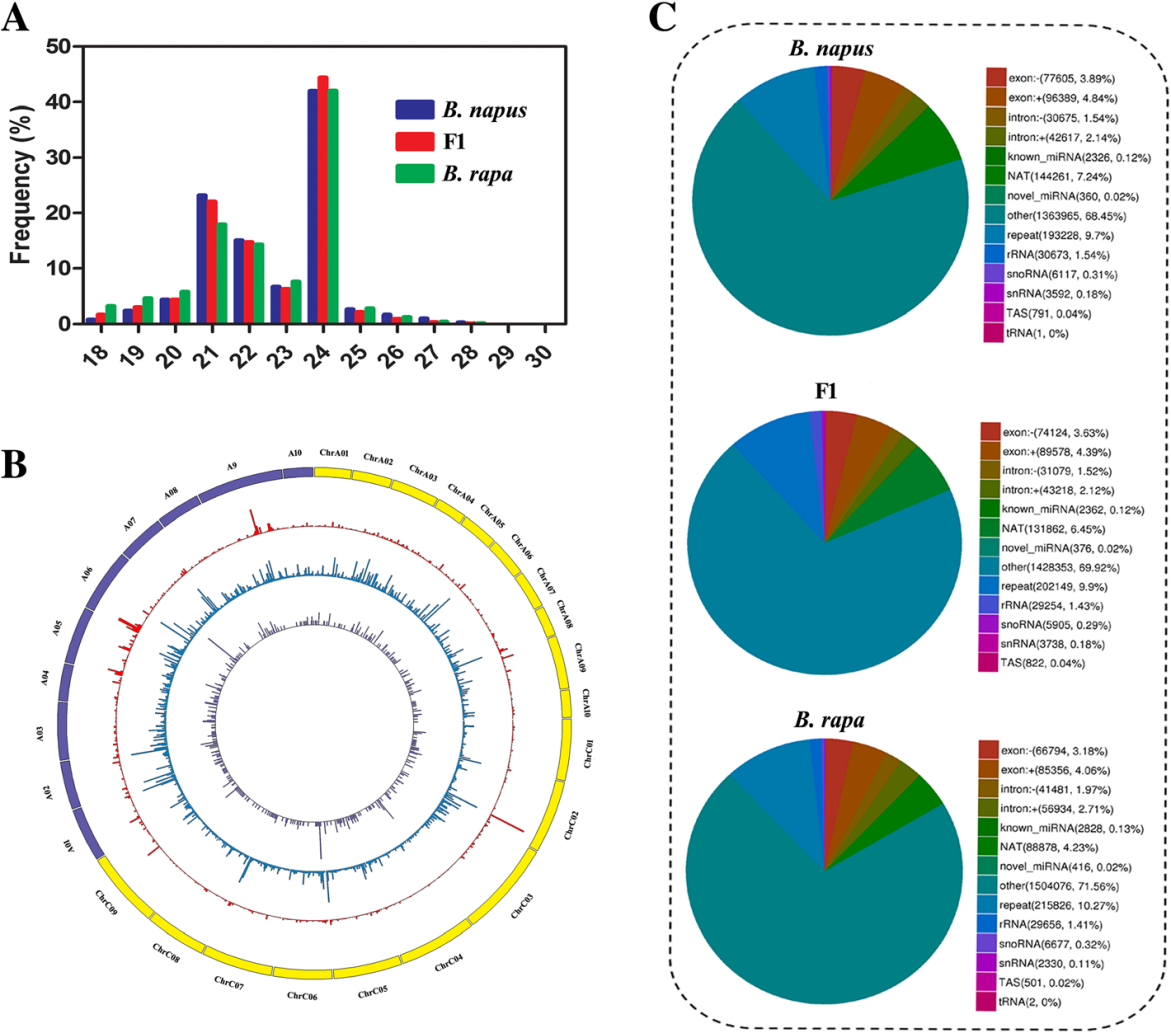
Bioinformatics analysis of small RNA sequencing. **a** The length distribution of small RNAs in *B. napus*, *B. rapa* and F1 hybrid. **b** MiRNA distribution in the genome of hybrid of *B. napus* and *B. rapa* hybrid. From outer to inter circles: (1) chromosomes of hybrid (Yellow indicates the chromosomes of *B. napus*; Purple indicates the chromosomes of *B. rapa*); (2) small RNA reads; (3) 24 nt small RNA reads; (4) mature miRNA reads. **c** The percentage of small RNAs of *B. napus*, *B. rapa* and F1 hybrid

Total RNA from developing embryos of *B. napus*, *B. rapa* and their F1 hybrid was isolated for the preparation of small RNA libraries. To investigate small RNA expression patterns in *B. napus*, *B. rapa* and their F1 hybrid, three biological replicates were sequenced using the Illumina Hi-Seq 2000 platform. After removing adaptor sequences and low-quality reads, ~ 15.2, 16.7 and 15.8 million clean reads were generated for *B. napus*, *B. rapa* and the F1 hybrid, respectively. The lengths of most clean reads were 21–24 nt according to statistical analysis (Fig. 1a). The richest small RNA reads were 24 nt and accounted for ~ 40% of the clean reads. Furthermore, we investigated the distribution of reads mapping on the chromosomes of *B. napus*, *B. rapa* and F1 hybrid genomes. As shown in Fig. 1b, Circos analysis showed that ~ 7.5 and 10.8 million clean reads mapped to the A and C genomes of *B. napus*, respectively. Additionally, we observed that ~ 15.7 million clean reads mapped to the A genome of *B. rapa*. As most of the small RNA reads were 24 nt, we examined the distribution of 24-nt clean reads and found that ~ 3.5 and 4.7 and 5.2 million clean reads of 24 nt mapped to the A and C genomes of *B. napus* and the A genome of *B. rapa*, respectively (Fig. 1b). Figure 1c shows that repeat RNA, NAT (Natural Antisense Transcript) RNA and intron sequences were the top 3 enriched types among all mapped small RNAs.

### Identification of conserved and novel miRNAs

**Fig. 2:**
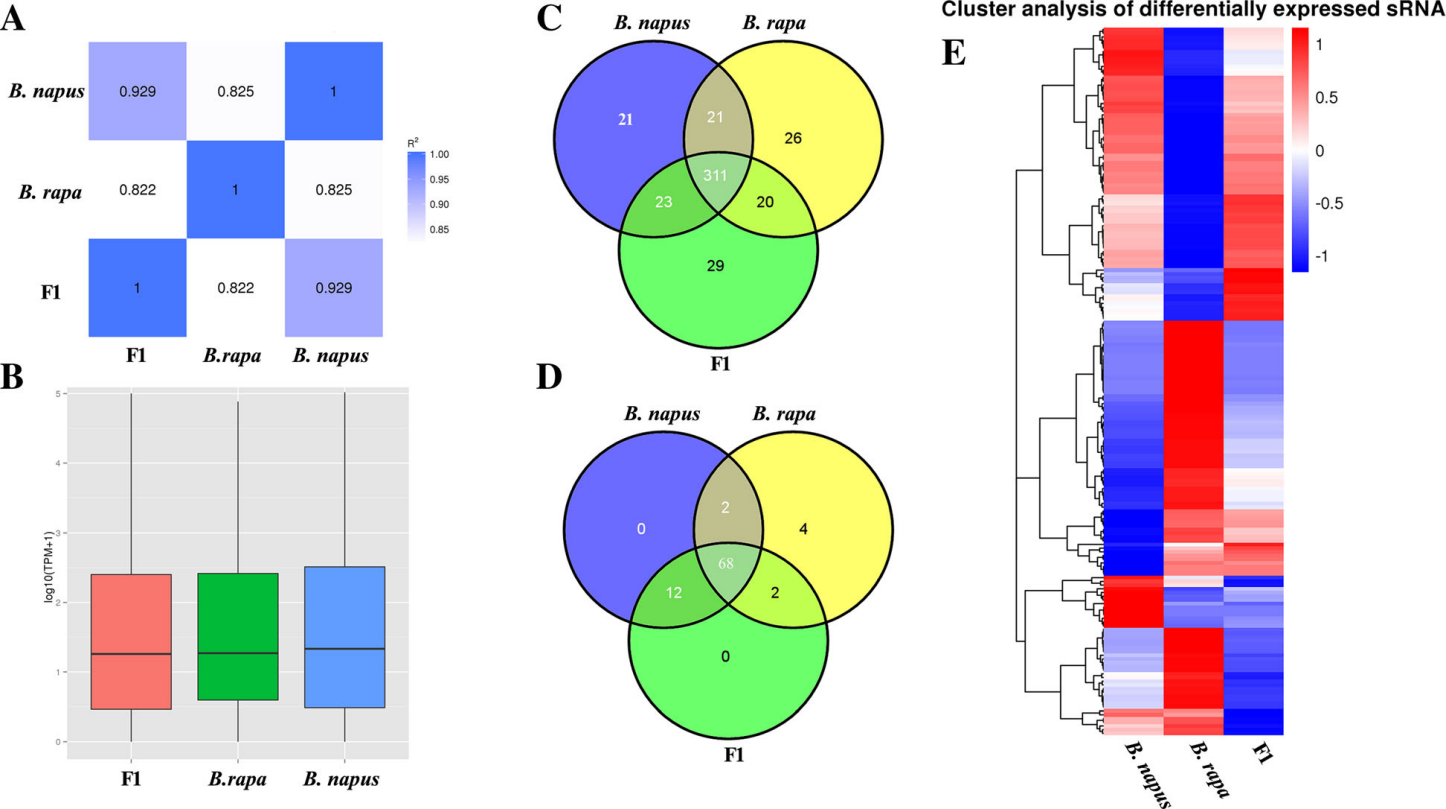
miRNA identification and differentially expressed miRNA analysis in *B. napus*, *B. rapa* and F1 hybrid. **a** The correlation analysis of the clean reads. **b** TPM distribution of the clean reads. **c** Venn diagram analysis of the identified conserved miRNAs in *B. napus*, *B. rapa* and F1 hybrid. **d** Venn diagram analysis of the identified novel miRNAs in *B. napus*, *B. rapa* and F1 hybrid. **e** Heatmap analysis of the differentially expressed miRNAs in *B. napus*, *B. rapa* and F1 hybrid

To identify conserved miRNAs in *B. napus*, *B. rapa* and the F1 hybrid, we first investigated correlation of the clean reads with TPM distribution in the *B. napus*, *B. rapa* and F1 hybrid samples. The correlation coefficients were 0.929, 0.822 and 0.825 between *B. napus* and the F1 hybrid, *B. rapa* and the F1 hybrid and *B. napus* and *B. rapa,* respectively (Fig. 2a). Additionally, the TPMs of *B. napus*, *B. rapa* and the F1 hybrid were mainly distributed around 1 and 2 (Log_10_^(TPM + 1)^) (Fig. 2b). We next mapped the unique clean reads to known miRBase miRNAs, and 451 conserved miRNAs were identified, including 376 miRNAs in *B. napus*, 378 miRNAs in *B. rapa* and 383 miRNAs in the F1 hybrid (Fig. 2c, Additional file 2: Table S2). Further analysis revealed the 451 conserved miRNAs to be distributed in 69 families, including miRNA156, miR159, miRNA156, and miRNA171_1 (Additional file 3: Table S3). We observed that 21, 26 and 29 miRNAs were specifically expressed in *B. napus*, *B. rapa* and the F1 hybrid, respectively. In addition, the expression abundance of conserved miRNAs among *B. napus*, *B. rapa* and F1 hybrid was quite variable. The expression level of ~ 40% of the conserved miRNAs was less than 10 reads; in contrast, we observed from 10 to 1000 reads for ~ 43% of the conserved miRNAs and more than 1000 reads for ~ 17% of the conserved miRNAs (Additional file 4: Table S4). Novel miRNAs among *B. napus*, *B. rapa* and the F1 hybrid were identified using published criteria [42], with 88 identified (Fig. 2d, Additional file 5: Table S5) including 82, 76 and 82 novel miRNAs in *B. napus*, *B. rapa* and the F1 hybrid, respectively. Statistical analysis showed that ~ 77% of 88 novel miRNAs (68 miRNAs) were shared by *B. napus*, *B. rapa* and the F1 hybrid. Moreover, 70 novel miRNAs were coexpressed between *B. napus* and *B. rapa*, 80 novel miRNAs were coexpressed between *B. napus* and the F1 hybrid, and 70 novel miRNAs were coexpressed between *B. rapa* and the F1 hybrid. We also observed that the expression abundance of some novel miRNAs in the F1 hybrid was much higher than the average expression level in its progenitors. For instance, novel_82 had 3-fold higher expression in the F1 hybrid than the average level in its progenitors. In addition, expression abundance analysis of novel miRNAs had an expression level of less than 10 reads for ~ 33% of the novel miRNAs; 10–1000 reads was found for ~ 62% of the novel miRNAs and more than 1000 reads for ~ 5%. To investigate the global miRNA expression profile, we performed hierarchical clustering of expressed miRNAs between the triploid hybrid and its inbred parents, and the results showed 379 miRNAs to be expressed in all three samples (Fig. 2c, d). Euclidean distances based on hierarchical clustering analysis between *B. napus* and the F1 hybrid, *B. rapa* and the F1 hybrid, and *B. napus* and *B. rapa* were 85.46, 59.78 and 48.66, respectively, which indicates that the expression profile of miRNAs of the F1 hybrid was more similar to that of *B. napus* than *B. rapa.* (Fig. 2e).

### Differential miRNA expression and miRNA target analysis

**Fig. 3:**
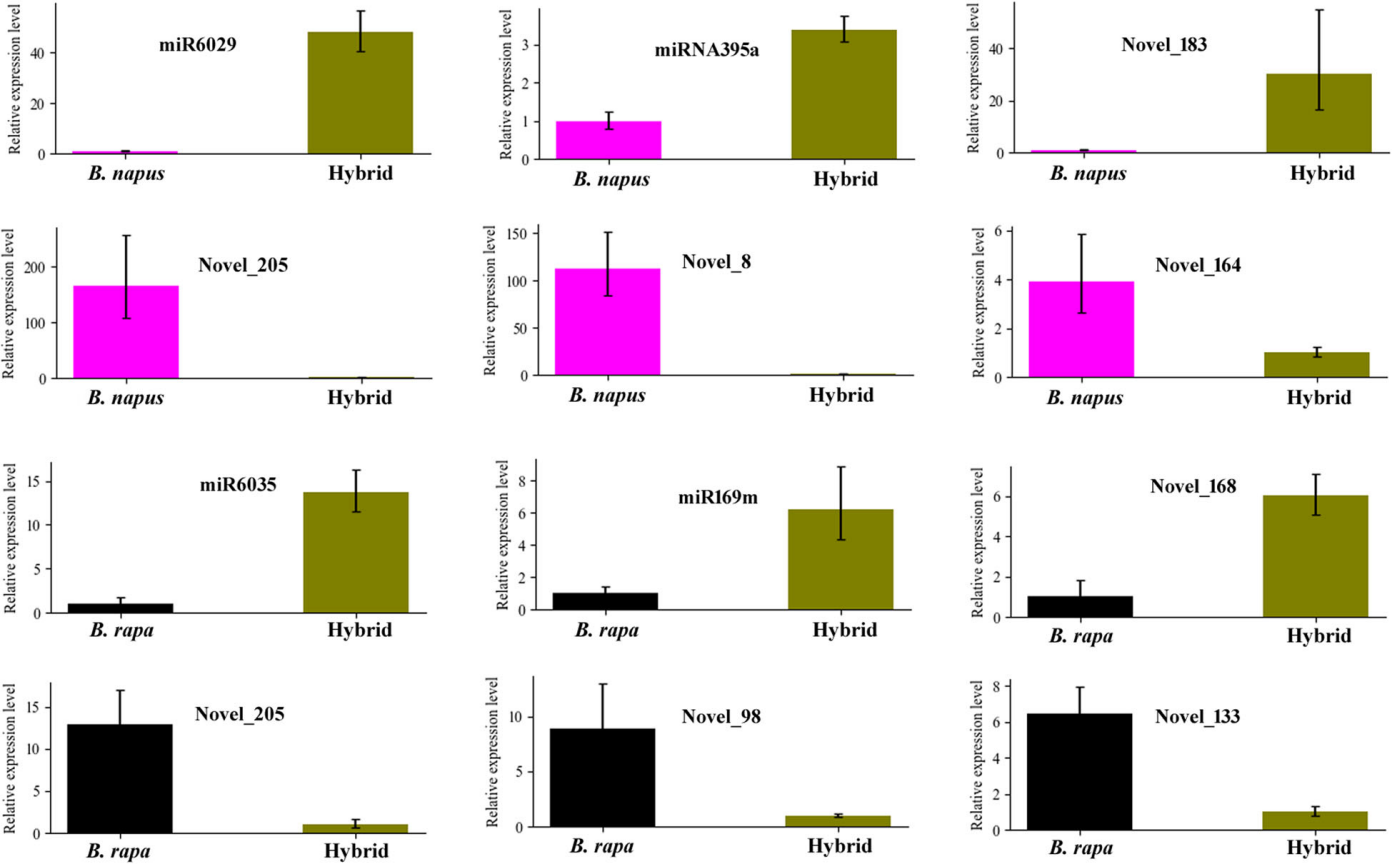
Quantitative RT-PCR Validation of differentially miRNAs between *B. napus*, *B. rapa* and the F1 hybrid

**Fig. 4:**
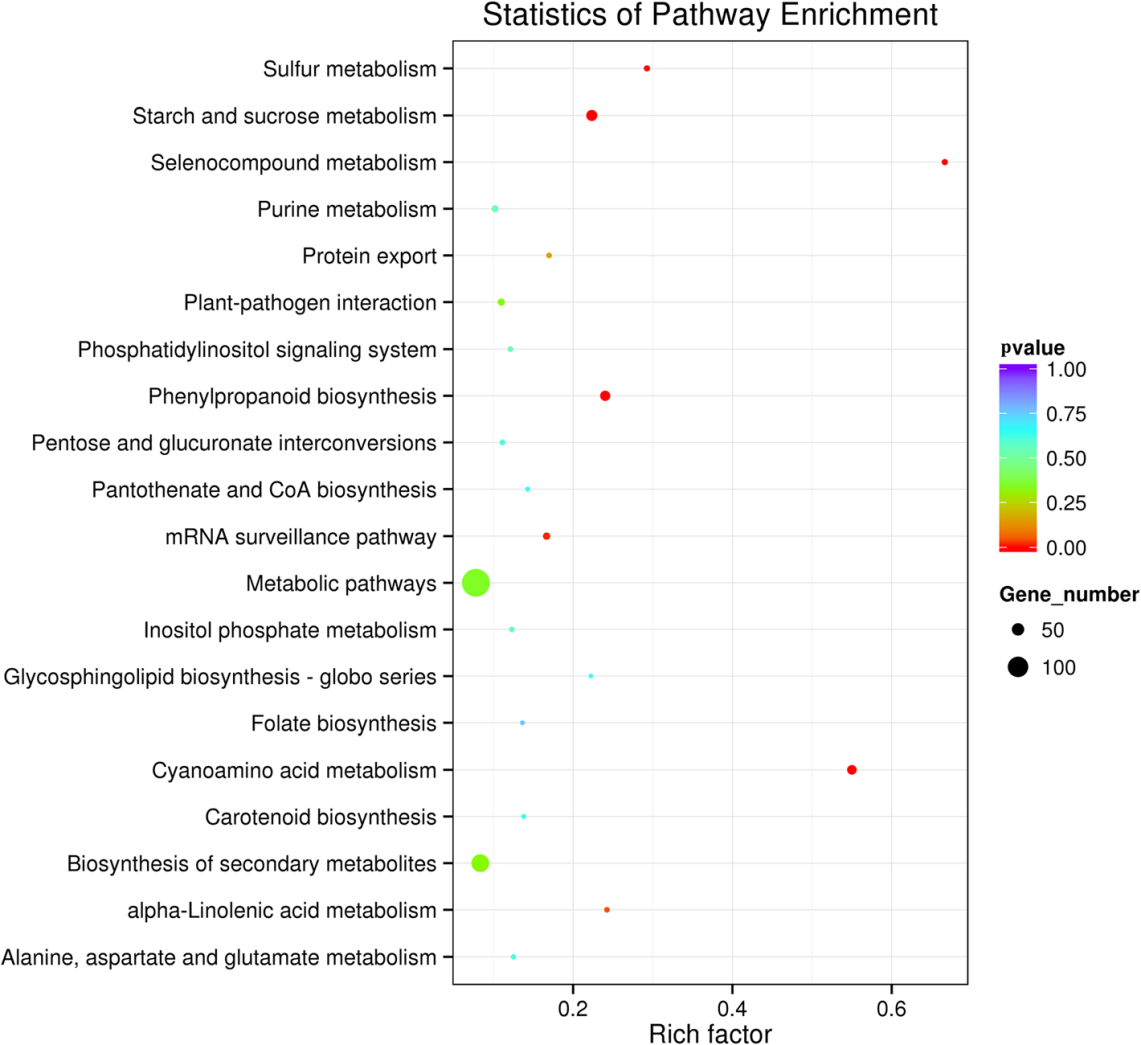
KEGG pathway analysis of the differentially expressed miRNAs in *B. napus*, *B. rapa* and F1 hybrid (Top 20 pathways pathways)

To investigate differentially expressed miRNAs between *B. napus*, *B. rapa* and the F1 hybrid, we analyzed the expression abundance of each miRNA by normalizing the read count to TPM (Fig. 2e). The results showed that a total of 213 miRNAs were differentially expressed between the *B. napus*, *B. rapa* and the F1 hybrid. Further analysis revealed 36 differentially expressed miRNAs (DE-miRNAs) between *B. napus* and the F1 hybrid, including 13 upregulated and 23 downregulated miRNAs (FDR *<* 0.001), respectively (Additional file 6: Table S6). Additionally, 155 DE-miRNAs were identified between *B. rapa* and the F1 hybrid, including 70 upregulated and 85 downregulated miRNAs, respectively (Additional file 6: Table S6). We also observed that 172 miRNAs were differentially expressed between *B. napus* and *B. rapa*, including 85 upregulated and 87 downregulated miRNAs, respectively (Additional file 6: Table S6). Collectively, more differentially expressed miRNAs were identified between *B. rapa* and the F1 hybrid or *B. napus* and *B. rapa*. To validate the differentially expressed miRNAs between *B. napus*, *B. rapa* and F1 hybrid, some miRNAs were selected for qRT-PCR analysis. Figure 3 showed that qRT-PCR experiment results were consistent with the small RNA sequencing data (Additional file 6: Table S6) and confirmed the differential expression of miRNAs between *B. napus*, *B. rapa* and F1 hybrid. The mRNA targets of the identified differentially expressed miRNAs among *B. napus*, *B. rapa* and the F1 hybrid were predicted using psRobot, which generated 9196 target genes for 276 miRNAs. All miRNA target genes were assigned Gene Ontology (GO) terms to examine the possible functions of these predicted miRNA target genes. These GO terms are distributed under three main categories: cellular component, biological process and molecular function. “Biological regulation”, “Regulation of cellular process” and “Regulation of biological process” were significantly enriched in the biological process category. “Cell”, “Cell part”, “Intracellular” and “Intracellular part” were significantly enriched in the cellular component category, and “Anion binding”, “Ribonucleotide binding”, “Nucleotide binding” and others were significantly enriched in the molecular function category. Furthermore, the predicted target genes of the miRNAs differentially expressed between the F1 hybrid and its progenitors were annotated using the KEGG database (ranked by *P*-value). The top 20 KEGG pathways are shown in Fig. 4. The top five pathways included “Cyanoamino acid metabolism” (33 genes, *P* = 5.68 × 10^− 15^), “Starch and sucrose metabolism” (42 genes, *P* = 1.68 × 10^− 8^), “Phenylpropanoid biosynthesis” (37 unigenes, *P* = 2.28 × 10^− 8^), “Selenocompound metabolism” (12 unigenes, *P* = 2.38 × 10^− 6^) and “Sulfur metabolism” (12 unigenes, *P* = 1.5 × 10^− 3^). As an essential process in plant growth and development, carbohydrate metabolism is closely related to biomass heterosis [48, 49]. We observed that various carbohydrate metabolism pathways, including “Cyanoamino acid metabolism”, “Starch and sucrose metabolism” and “Phenylpropanoid biosynthesis”, were significantly enriched among the target genes of differentially expressed miRNAs between *B. napus*, *B. rapa* and the F1 hybrid. Because these differentially expressed miRNAs mainly clustered in miR156, miR159 and miR166, miR167, miR171_1, miR395, miRNA396, and miR399 families, we conclude that these differentially expressed miRNAs might play important roles in biomass heterosis by controlling expression of target genes in the hybrid of *B. napus* and *B. rapa.*

**Fig. 5:**
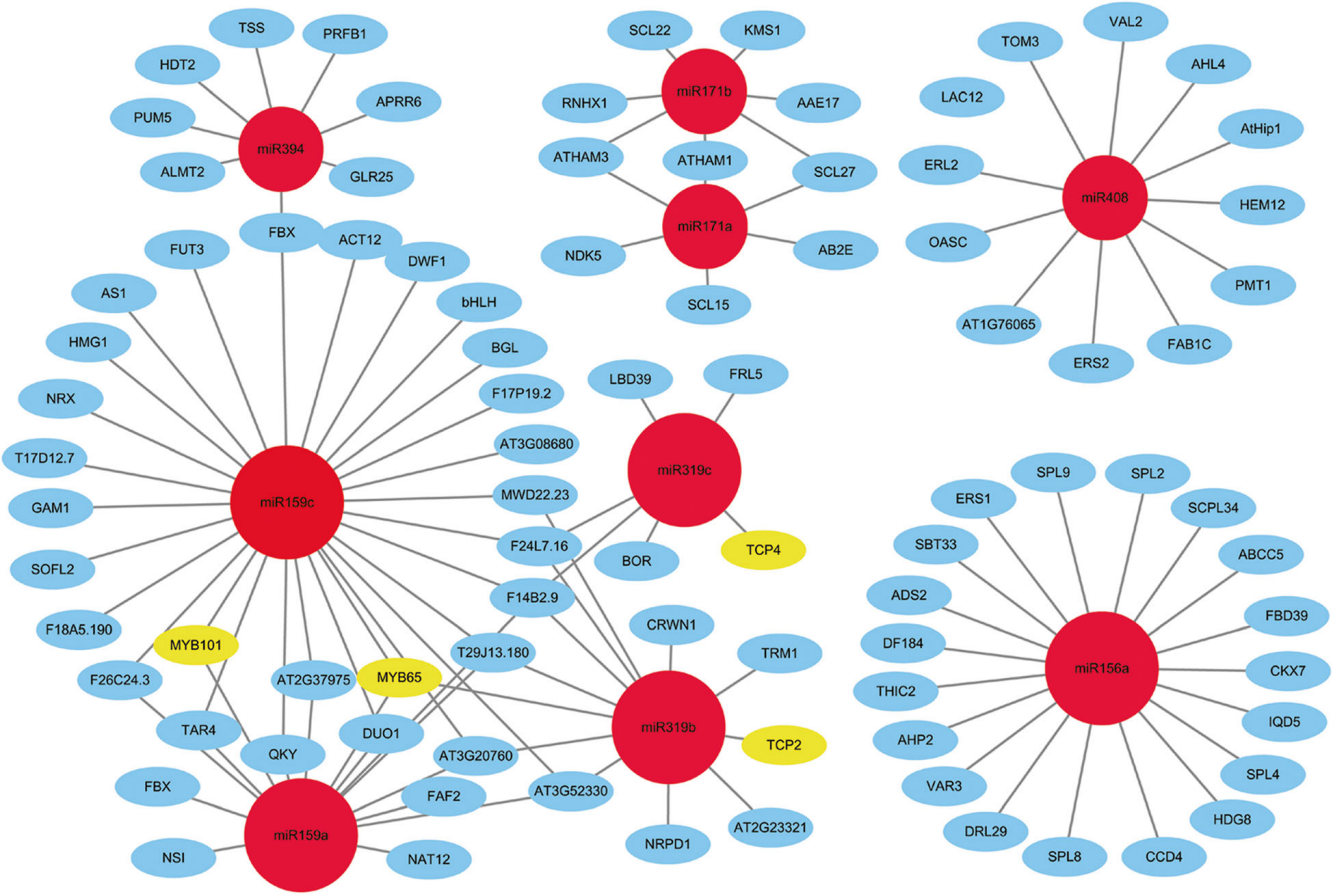
MiRNA-mRNA interaction network analysis in *B. napus*, *B. rapa* and F1 hybrid (Red indicates the miRNAs; Blue indicates the target genes; Yellow indicates the important transcription factors)

Furthermore, we investigated the interaction network between miRNAs and their mRNA targets (Fig. 5). For instance, miR159c and miR156a and miR159a target a large number of genes in miRNA-mRNA interaction networks, suggesting that these three miRNAs might be the key regulators of multiple mRNA targets. As shown in Fig. 5, MYB65 and MYB101, common mRNA targets of miR159c and miR159a, are reported to be essential transcription factors in various plant development processes [50, 51]. Moreover, Fig. 5 indicates that ERS2 is one of the mRNA targets of miR408. A previous study showed that ERS2 is involved in the ethylene-activated signaling pathway and plays many vital roles in plant growth and development [52]. Taken together, these results indicate that the target genes are linked to development and metabolism processes in the F1 hybrid and progenitors, which suggests essential roles of miRNA in allopolyploidization and heterosis.

### Non-additive miRNA expression analysis

**Fig. 6:**
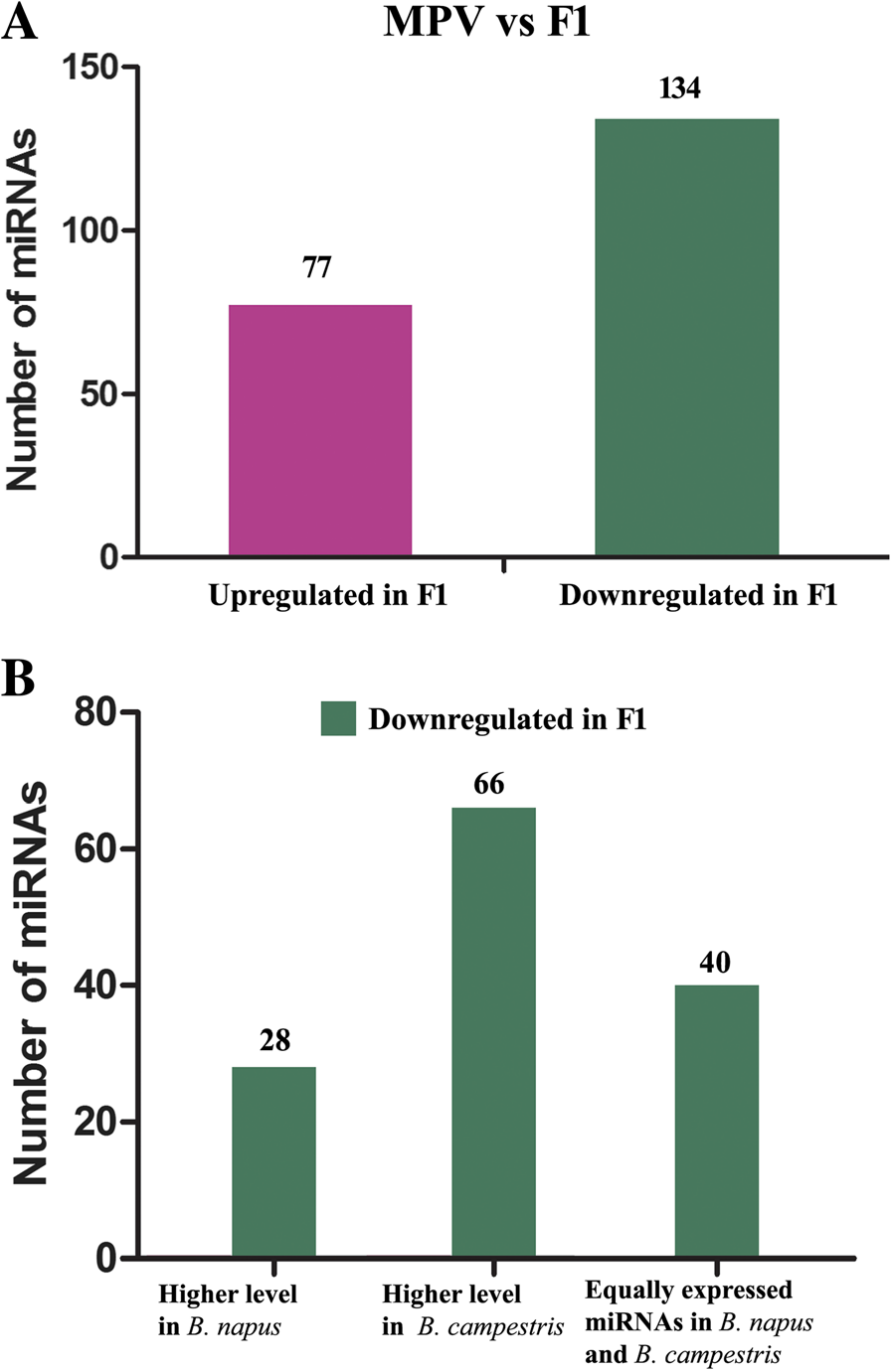
Non-additive miRNA expression analysis between *B. napus*, *B. rapa* and F1 hybrid. **a**. The number of upregulated and downregulated non-additively expressed miRNAs in F1 hybrid. **b**. Expression pattern analysis of 134 down-regulated miRNAs in F1 hybrid

To investigate how polyploidization affects miRNA expression patterns, we performed non-additive miRNA expression analysis, as previously described [53]. In brief, the miRNA expression values for the F1 hybrid were compared with the average miRNA expression values for *B. napus* and *B. rapa*, and miRNAs were identified as being nonadditively expressed if the fold change was greater than 2 (*P* ≤ 0.05) in comparison with the MPV (mid-parent value), the average miRNA expression value for *B. napus* and *B. rapa*. The results showed that 211 miRNAs, including 77 up- and 134 downregulated miRNAs, were nonadditively expressed in the F1 hybrid (Fig. 6a). Importantly, more downregulated miRNAs than upregulated miRNAs (134 vs 77) were nonadditively expressed in the F1 hybrid, which is consistent with previous nonadditive analysis in *Brassica* hexaploidy [53]. We further analyzed the expression patterns of the 134 downregulated miRNAs in the F1 hybrid and found that 28 miRNAs with higher expression levels in *B. napus* than in *B. rapa* were downregulated in the F1 hybrid (Fig. 6b). Moreover, 66 miRNAs with higher expression in *B. rapa* than in *B. napus* were downregulated in the F1 hybrid, and 40 miRNAs with higher equal expression between *B. napus* and *B. rapa* were downregulated in the F1 hybrid. Taken together, our data indicate that more miRNAs with higher expression in *B. rapa* than in *B. napus* are repressed in the F1 hybrid.

### MiRNA synteny analysis in allopolyploidization

**Fig. 7:**
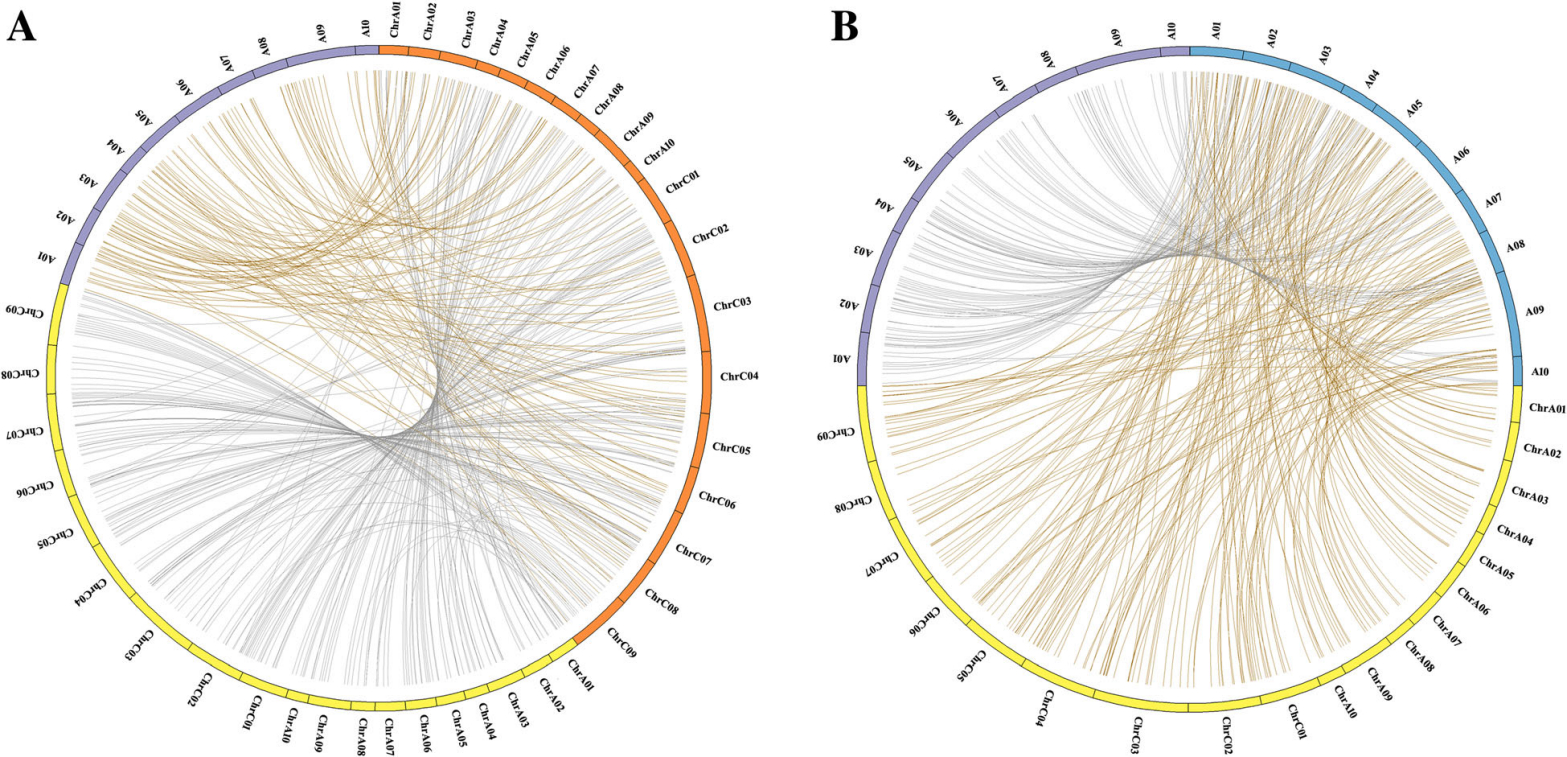
MiRNA synteny analysis between F1 hybrid (A^n^A^r^C^n^) and two progenitors A^n^A^n^C^n^C^n^ and A^r^A^r^. The miRNAs of A^n^A^n^C^n^C^n^ and A^r^A^r^ were mapped to F1 genome. The lines indicate the matches of miRNAs from two progenitors to F1 genome. **a**. Yellow indicates the A^n^ and C^n^ genomes of F1 hybrid; Purple indicates the A^r^ genome of F1 hybrid; Orange indicates the *B. napus* genome. **b**. Yellow indicates the A^n^ and C^n^ genomes of F1 hybrid; Purple indicates the A^r^ genome of F1 hybrid; Blue indicates the *B. rapa* genome

*Brassica* species are good models for studying genome expansion and gene fractionation in polyploids [54–58]. The available genome sequences of *B. napus* and *B. rapa* are very helpful for investigating miRNA expansion and loss in the hybrid of *B. napus* and *B. rapa*. We first performed miRNA synteny analysis between *B. napus*, *B. rapa* and the F1 hybrid using the predicted miRNAs and their flanking sequences. As shown in Figs. 7, 95.5% of the *B. napus* and *B. rapa* miRNAs (715/749) are present in the genome of the F1 hybrid (Additional file 7: Table S7). Among them, 350 *B. rapa* miRNAs and 365 *B. napus* miRNAs mapped to the A^r^ and A^n^C^n^ genomes of the F1 hybrid, respectively. We also observed some miRNAs to be located on the opposite genome of the F1 hybrid. For example, 238 *B. rapa* miRNAs are located on the A^n^C^n^ genome and 135 *B. napus* miRNAs on the A^r^ genome in the F1 hybrid (Additional file 7: Table S7). Overall, the miRNA synteny analysis results indicated high conservation between the A^n^A^n^C^n^C^n^, A^r^A^r^ genome and the A^n^A^r^C^n^ genome of the F1 hybrid. However, we did find that 74 miRNAs, including 68 conserved miRNAs and 6 novel miRNAs, have no homology with the A^n^A^r^C^n^ genome of the F1 hybrid. Comparative genome analysis between *B. napus*, *B. rapa* and their triploid hybrid revealed that some conserved miRNAs in miR166 and miR399 families have been lost from the A^n^A^r^C^n^ genome of the F1 hybrid, possibly due to gene fractionation and mutation. Furthermore, our results showed that 34 miRNAs, including 30 conserved miRNAs and 4 novel miRNAs (Additional file 7: Table S7), are not present in the syntenic regions of the A^n^A^n^C^n^C^n^ and A^r^A^r^ genomes, which indicates that these miRNAs may have been generated after the allopolyploidization event in the F1 hybrid. We hypothesize that these newly generated miRNAs were induced by random DNA segmental duplication or insertions in the F1 genome and that they are related to environmental adaptation under specific selection pressure. Taken together, the results indicate a certain amount of miRNA loss and insertions in the genome of the F1 hybrid in comparison with its progenitors *B. napus* and *B. rapa*.

## Discussion

Polyploidy is an important process of plant evolution. Interspecific hybridization usually results in hybrid vigor, disease resistance and some other traits in plant allopolyploids [59–61]. In the process of polyploid production, genetic and epigenetic effects are stimulated by distant hybridization and polyploidy. Various studies have shown that miRNAs play essential roles in the genomic interaction of many plant hybrids [14, 21, 62]. For instance, miRNA expression will increase, and transposons will be activated in interspecific hybrids, which will alter gene expression in the new polyploid and allow for adaptation to environmental changes [14, 19].

In this study, we used high-throughput sequencing technology to globally identify miRNAs, including conserved and novel miRNAs, in *B. napus, B. rapa* and their F1 hybrid. A total of 451 conserved miRNAs were identified, including 376, 378 and 383 miRNAs in the *B. napus*, *B. rapa* and F1 hybrid, respectively. In addition, 88 novel miRNAs were identified in *B. napus*, *B. rapa* and the F1 hybrid. Bioinformatics analysis showed that the expression level of these novel miRNAs was lower than that of conserved miRNAs, which suggested some important roles for these novel miRNAs in *B. napus*, *B. rapa* and the F1 hybrid. Moreover, bioinformatics analysis of differentially expressed miRNAs indicated that the genome-wide miRNA expression pattern of the F1 hybrid was more similar to that of *B. napus* than *B. rapa,* indicating that miRNAs play important roles in the genomic interaction of plant hybrids.

A total of 9196 target genes of the differentially expressed miRNAs were predicted by psRobot. Gene Ontology analysis showed that the target genes are mainly associated with “biological regulation”, “regulation of cellular process” and “regulation of biological process” in the biological process category. More importantly, we observed that some predicted miRNA target genes are important transcription factors (TFs), including TCP2, TCP4, MYB65 and MYB101 (Fig. 5). TCP transcription factors regulate the growth and development of leaves and flowers [63]. Moreover, miR319 affects the formation of leaf shape and flowering time by specifically inhibiting expression of TCP mRNAs [64]. In addition, a previous study showed that miR159 overexpression inhibits expression of MYB transcription factors and leads to male sterility [65]. Taken together, compared with the roles of miRNAs in other plants, the miRNAs differentially expressed between the F1 hybrid and its progenitors might be potential determinants in the heterosis of the *B. napus* and *B. rapa* hybrid.

In polyploidy, nonadditive miRNA expression is usually induced by interspecific hybridization, resulting in gene expression and phenotype complexity. For example, comparative analysis between *Arabidopsis thaliana*, *A. arenosa* and their hybrid offspring showed that miRNAs are highly conserved among the species and that a large number of are are nonadditively expressed between the interspecific hybrid and its progenitors [14]. In particular, comparative analysis between a hexaploid hybrid and its parents showed 68.8% of miRNAs to be nonadditively expressed. Moreover, most of the nonadditively expressed miRNAs were inhibited and displayed similar expression patterns in *B. rapa* and hexaploid hybrids [53]. Our study showed that more downregulated miRNAs (134 miRNAs) than upregulated miRNAs (77 miRNAs) were nonadditively expressed in the F1 hybrid, which is consistent with a previous study investigating hexaploidy in *Brassica* [53]. Our synteny analysis allowed exact miRNA localization in orthologous regions of the genome in the F1 hybrid, and the results showed that the *B. napus* and *B. rapa* genomes and F1 genome share very high homology. Nevertheless, no homology of 74 *B. napus* and *B. rapa* miRNAs was found for the F1 hybrid, suggesting that some miRNAs in *B. napus* and *B. rapa* have been lost from the F1 hybrid genome, possibly due to DNA fragment deletions and mutations. We also observed that 34 miRNAs in the F1 hybrid were not present in the syntenic regions of the *B. napus* and *B. rapa* genomes, indicating that these miRNAs might have been generated after the allopolyploidization event by random DNA segmental duplication or insertions in the F1 genome. Taken together, this study not only provides a useful resource to explore global miRNA expression patterns and evolution and genome structure in allopolyploid plants but also offers a promising perspective on the mechanism of miRNA regulation in allopolyploids, and the findings promote the genetic studies of genomic interaction in allopolyploids.

## Conclusions

This study provides fundamental expression patterns of conserved and novel miRNAs between *B. napus*, *B. rapa* and F1 hybrid. Bioinformatics analysis showed that 376, 378 and 383 conserved miRNAs, as well as 82, 76 and 82 novel miRNAs, were identified in *B. napus*, *B. rapa* and F1 hybrid, respectively. Moreover, 213 miRNAs were found to be differentially expressed between *B. napus*, *B. rapa* and F1 hybrid. Meanwhile, the present study showed that a total of 211 miRNAs, including 77 up-regulated and 134 downregulated miRNAs, were non-additively expressed in F1 hybrid. Furthermore, miRNA synteny analysis revealed a high miRNA genomic conservation between the genomes of *B. napus*, *B. rapa* and their F1 hybrid genome, and some miRNA loss and gain events in the genome of F1 hybrid. This study provided useful resources to explore global miRNA expression pattern and genome structure, which will be very helpful to reveal the miRNA-mediated regulatory mechanisms in the genomic interactions of *Brassica* allopolyploid.

## Additional files

Additional file 1: **Table S1.** Primers for qRT-PCR validation of differentially expressed miRNAs between *B. napus*, *B. rapa* and the triploid hybrid. (DOCX 15 kb)

Additional file 2: **Table S2.** The identified conserved miRNAs in *B. napus*, *B. rapa* and F1. (XLSX 23 kb)

Additional file 3: **Table S3.** The identified miRNA families in *B. napus*, *B. rapa* and F1. (XLSX 35 kb)

Additional file 4: **Table S4.** The expression level of the conserved miRNAs in *B. napus*, *B. rapa* and F1. (XLSX 23 kb)

Additional file 5: **Table S5.** The identified novel miRNAs in *B. napus*, *B. rapa* and F1. (XLSX 13 kb)

Additional file 6: **Table S6.** The differentially expressed miRNAs between *B. napus*, *B. rapa* and F1. (XLSX 12 kb)

Additional file 7: **Table S7.** The miRNA synteny analysis of *B. napus*, *B. rapa* and F1. (XLSX 30 kb)

## Data Availability

The sequencing data of *B. napus*, *B. rapa* and F1 hybrid in this study were deposited in NCBI Sequence Read Archive (SRA) Sequence Database with accession number SRR9332479, SRR9332480, SRR9332481, SRR9332482, SRR9332475, SRR9332476, SRR9332477, SRR9332478, SRR9332483.

## Abbreviations

*A. thaliana*: *Arabidopsis thaliana*
*B. napus*: *Brassica napus*
*B. rapa*: *Brassica rapa*
DE-miRNA: Differentially expressed miRNA
miRNA: MicroRNA
TFs: Transcription factors.
TPM: Transcripts per million
