# Supplementary material for: Characterization and expression profiles of miRNAs in the triploid hybrids of Brassica napus and Brassica rapa
Source: BMC Genomics. 2019 Aug 14;20:649. doi: 10.1186/s12864-019-6001-x (PMC6694508; doi:10.1186/s12864-019-6001-x)
Supplement: Supplementary file 1 — Table S1. Primers for qRT-PCR validation of differentially expressed miRNAs between B. napus, B. rapa and the triploid hybrid. (DOCX 15 kb) [file 12864_2019_6001_MOESM1_ESM.docx]

| **Table S1: Primers for qRT-PCR validation of differentially expressed miRNAs between *B. napus, B. rapa and the triploid hybrid*.** | | |
| --- | --- | --- |
| miR6029 | CGCGTGGGGTTGTGATTTC | GTCGTATCCAGTGCAGGGTCCGAGGTATTCGCACTGGATACGACAAGCCT |
| miR395a | GCGCTGAAGTGTTTGGGG | GTCGTATCCAGTGCAGGGTCCGAGGTATTCGCACTGGATACGACGAGTTC |
| miR6035 | GCGCGTGGAGTAGAAAATGC | GTCGTATCCAGTGCAGGGTCCGAGGTATTCGCACTGGATACGACACGACT |
| miR169m | CGCGTGAGCCAAAGATGAC | GTCGTATCCAGTGCAGGGTCCGAGGTATTCGCACTGGATACGACCGGCAA |
| Novel_183 | GCGCGATCTCTATTTTTACCTCT | GTCGTATCCAGTGCAGGGTCCGAGGTATTCGCACTGGATACGACTATTTT |
| Novel_205 | CGAATTCCGACGGAAACAGC | GTCGTATCCAGTGCAGGGTCCGAGGTATTCGCACTGGATACGACGAACTA |
| Novel_8 | CGCGCTTTGCCTATCGTTT | GTCGTATCCAGTGCAGGGTCCGAGGTATTCGCACTGGATACGACTTTTCC |
| Novel_164 | CGATTTTGTCCCGGGAGG | GTCGTATCCAGTGCAGGGTCCGAGGTATTCGCACTGGATACGACCCATCA |
| Novel_168 | GCGCGACTTTGAAACTTTGA | GTCGTATCCAGTGCAGGGTCCGAGGTATTCGCACTGGATACGACTCTAGA |
| Novel_98 | CGCGCGATATTGGTACGGT | GTCGTATCCAGTGCAGGGTCCGAGGTATTCGCACTGGATACGACGATTCA |
| Novel_133 | GCGATCCTCGGGACACAG | GTCGTATCCAGTGCAGGGTCCGAGGTATTCGCACTGGATACGACGGTAAT |
| univ-R | AGTGCAGGGTCCGAGGTATT | |
| U6 | TTGGAACGATACAGAGAAGATTAGCA | TTGGACCATTTCTCGATTTGTG |
